# O-GlcNAcylation in Hyperglycemic Pregnancies: Impact on Placental Function

**DOI:** 10.3389/fendo.2021.659733

**Published:** 2021-06-01

**Authors:** Jie Ning, Huixia Yang

**Affiliations:** ^1^ Department of Obstetrics and Gynaecology, Peking University First Hospital, Beijing, China; ^2^ Beijing Key Laboratory of Maternal Foetal Medicine of Gestational Diabetes Mellitus, Beijing, China; ^3^ Peking University, Beijing, China

**Keywords:** O-GlcNAcylation, hyperglycemia in pregnancy, placental function, O-GlcNAc transferase, O-GlNAcase

## Abstract

The dynamic cycling of *N*-acetylglucosamine, termed as O-GlcNAcylation, is a post-translational modification of proteins and is involved in the regulation of fundamental cellular processes. It is controlled by two essential enzymes, O-GlcNAc transferase and O-GlcNAcase. O-GlcNAcylation serves as a modulator in placental tissue; furthermore, increased levels of protein O-GlcNAcylation have been observed in women with hyperglycemia during pregnancy, which may affect the short-and long-term development of offspring. In this review, we focus on the impact of O-GlcNAcylation on placental functions in hyperglycemia-associated pregnancies. We discuss the following topics: effect of O-GlcNAcylation on placental development and its association with hyperglycemia; maternal-fetal nutrition transport, particularly glucose transport, *via* the mammalian target of rapamycin and AMP-activated protein kinase pathways; and the two-sided regulatory effect of O-GlcNAcylation on inflammation. As O-GlcNAcylation in the placental tissues of pregnant women with hyperglycemia influences near- and long-term development of offspring, research in this field has significant therapeutic relevance.

## Introduction

Hyperglycemia in pregnancy (HIP), one of the most common medical conditions during pregnancy, may be classified as gestational diabetes mellitus (GDM) and diabetes mellitus in pregnancy. HIP is an important cause of adverse pregnancy outcomes and increasing incidences of metabolic syndromes in adulthood (1–3). The placenta is a key interface for maternal-fetal interaction, particularly for nutrition transport. It is instrumental in fetal intrauterine growth and long-term development of offspring. The placenta of women with HIP is exposed to a high concentration of blood glucose at different degrees and windows of time. This may affect numerous cellular pathways, leading to accumulation of advanced glycation end-products (4, 5) and induction of oxidative stress (6). The reported activation of the chronic hexosamine biosynthetic pathway (HBP) in placental tissue under similar conditions is also garnering attention (7).

**Figure 1:**
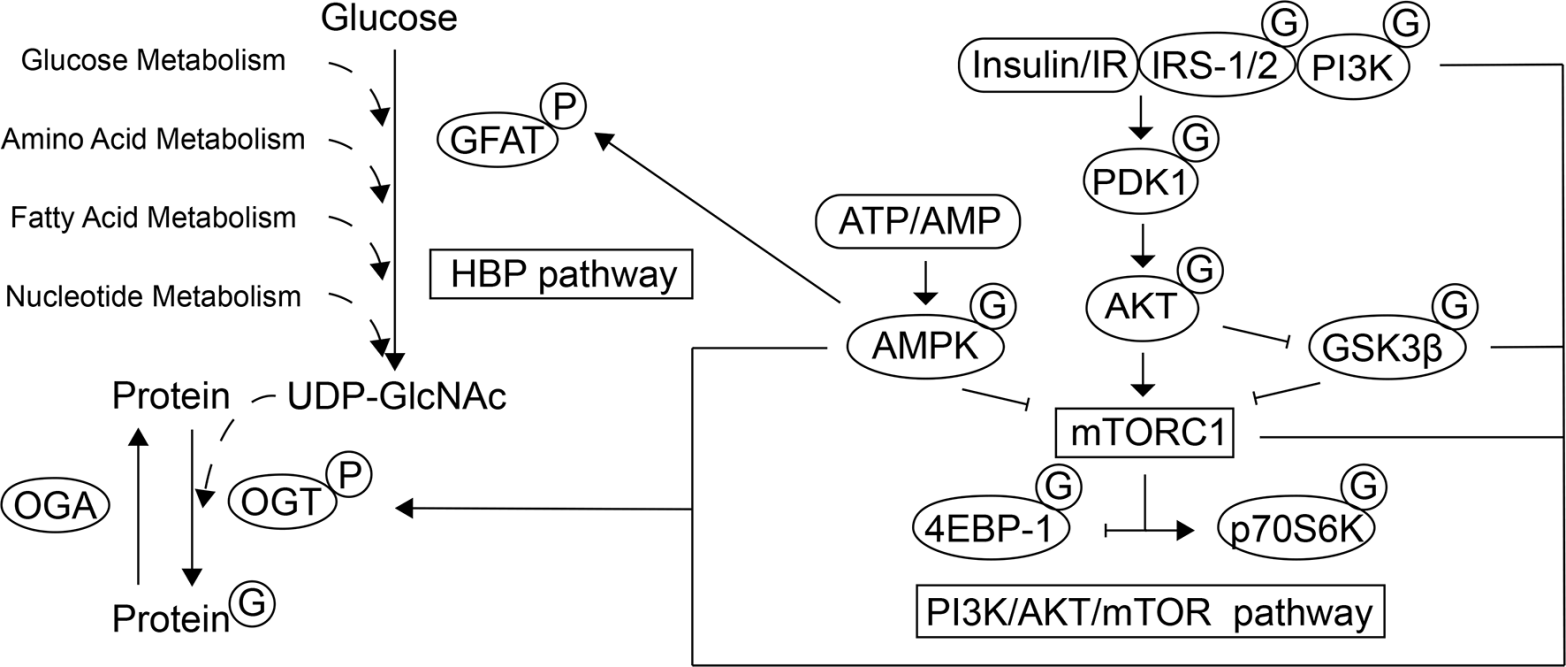
Schematic representation of the interplay between the HBP, mTOR and AMPK pathways. O-GlcNAcylation, as a PTM of a wide variety of nuclear, cytoplasmic, and mitochondrial proteins, participates in various cellular processes. The HBP integrates glucose, fatty acid, amino acid, and nucleotide metabolism to generate the substrate for O-GlcNAcylation, UDP-GlcNAc. GFAT is the rate-limiting step of the HBP and its activity can be regulated by AMPK through phosphorylation. The O-GlcNAc moiety can be transferred to the target proteins and removed by OGT and OGA, respectively. The activity of mTOR and GSK3β can be mediated by the PI3K/AKT signaling pathway and mTORC1 promotes protein synthesis by directly phosphorylating 4E-BP1 and p70S6K. In response to energy state, AMPK also regulates the mTORC1 activity. The localization, activity, and substrate specificity of OGT are regulated through phosphorylation by AMPK, IR/PI3K and GSK3β, and the mTOR signaling regulates the expression of OGT. In contrast, several actors of the PI3K/AKT/mTOR signaling pathway have been found to be modified by O-GlcNAcylation, which leads to subsequent biological effects under different physiological conditions.

O-linked β-N-acetylglucosamine (O-GlcNAc) glycosylation (O-GlcNAcylation) is a post-translational modification (PTM) of proteins that plays an essential role in regulating various cellular processes (**Figure 1**). In contrast to classical N-/O-linked glycosylation, which mostly occurs in the Golgi compartment and endoplasmic reticulum with the extraordinarily extracellular complex array of glycans, the substrate for O-GlcNAcylation is uridine diphosphate N-acetylglucosamine (UDP-GlcNAc) generated from HBP. HBP is a pathway that integrates glucose, fatty acid, amino acid, and nucleotide metabolism. The GlcNAc moiety from UDP-GlcNAc can be transferred onto the serine and threonine residues of a wide variety of nuclear, cytoplasmic, and mitochondrial proteins through the catalytic activity of the enzyme O-GlcNAc transferase (OGT). The cleavage of O-GlcNAc from proteins is catalyzed by glycoside hydrolase O-GlcNAcase (OGA) (also named MGEA5). Similar to other PTMs, this process is dynamic and reversible (7).

It has been reported that O-GlcNAcylation occurs in the placenta and is involved in transcriptional regulation, signal transduction, and epigenetic modifications (8–11). OGA is expressed in most tissues, and one of the highest expression was found in the placenta (12). OGT acts as a placental biomarker of maternal stress, which affects fetal neurodevelopment (13). Studies in diabetes mellitus have shown that hyperglycemia directly increases protein O-GlcNAcylation, at least in part, by increasing the glucose flux through HBP, and that OGT/OGA expression may be regulated by chronic hyperglycemia (14). Studies on hyperglycemic rat models have also shown that O-GlcNAcylation levels increase in the placenta depending on the severity of hyperglycemia, and that trophoblast cells were the main target for O-GlcNAcylation (8). The focus of this review is to summarize the impact of O-GlcNAcylation in placenta exposed to HIP.

## Placenta Growth and Development

**Figure 2:**
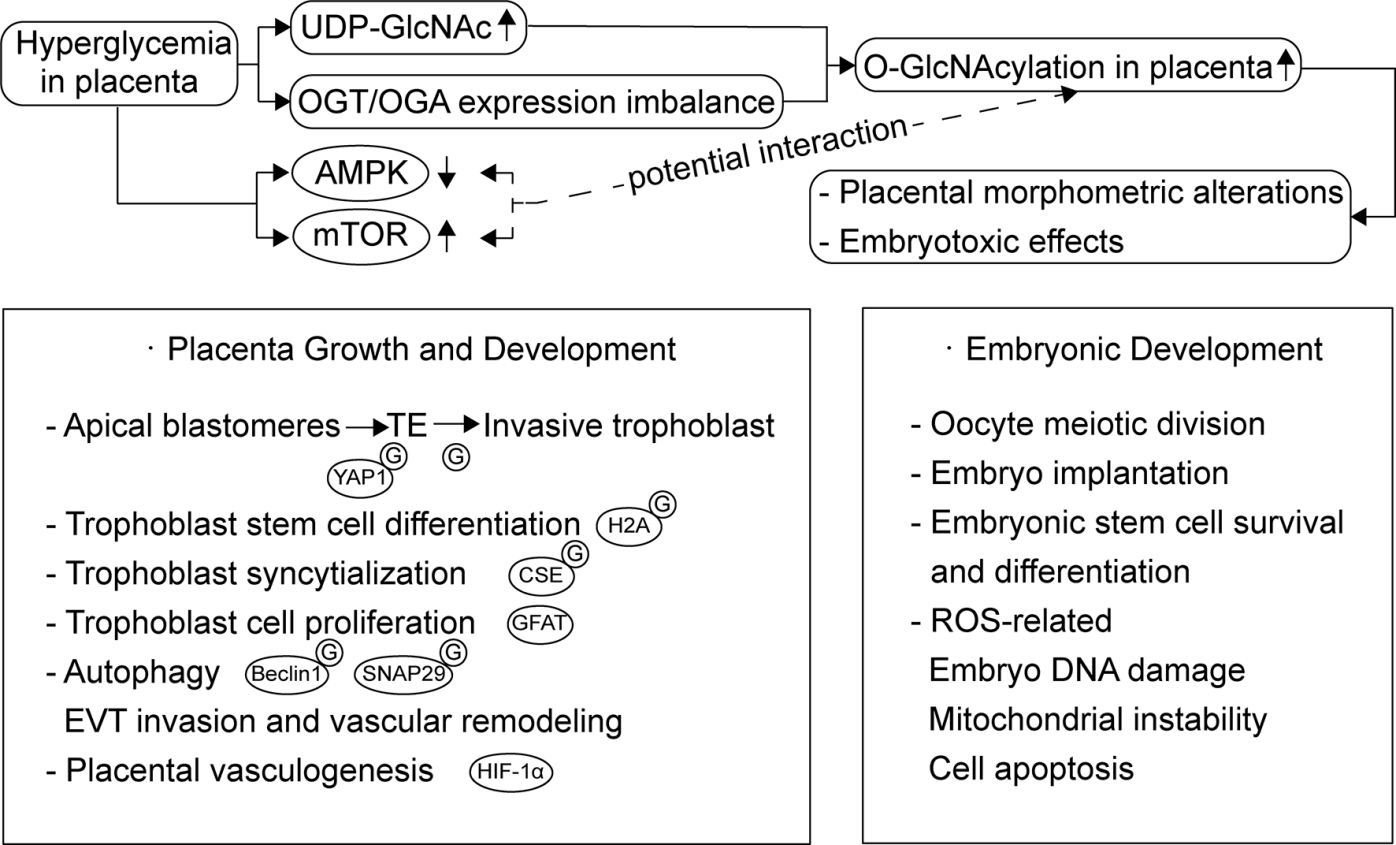
Overview of the function of O-GlcNAcylation during placentation and placental development and O-GlcNAcylation dysregulation in placenta exposed to HIP. Besides embryonic development, O-GlcNAcylation also plays a role through all stages of placental development, including trophoblast cell proliferation, differentiation, syncytialization and autophagy. Hyperglycemia increases the glucose flux through HBP and affects placental OGA expression, which leads to increased O-GlcNAcylation accumulation in placenta. These changes may result in placental morphometric alterations and embryotoxic effects. mTOR and AMPK activity altered in HIP and their potential interaction with O-GlcNAcylation needs further studies.

The placenta is involved in the development, adaptation, and physiology of offspring in response to maternal growth and nutrient signals, primarily by regulating nutrient transport. O-GlcNAcylation seems to be an important modulator during placentation and placental development (15) (**Figure 2**). Studies on mouse embryos have demonstrated that the nuclear localization of Yes-associated protein 1 (YAP1) is glucose/HBP/O-GlcNAcylation-dependent, and this event is crucial for differentiation of the apical blastomeres to form the extraembryonic trophectoderm (TE) (16). During the incipient stages of trophoblast development at implantation, Ruane et al. (17) proposed that O-GlcNAcylation drives TE differentiation to the invasive trophoblast, as well as the differentiation of BeWo to syncytiotrophoblasts (STBs). Moreover, the O-GlcNAcylation of histone variant H2A was also shown to participate in the trophoblast stem cell differentiation process (18). A recent study on the placenta suggested that the O-GlcNAcylation of cystathionine γ-lyase (CSE) at Ser138 promotes its activity to produce H2S. Further, H2S inhibits androgen receptor dimerization and then represses trophoblast syncytialization (19). Glutamine fructose-6-phosphate amidotransferase (GFAT), an important rate-limiting enzyme of the HBP, regulates trophoblast cell proliferation in response to glucose through phosphatidylinositol 3-kinase (PI3K)-independent mammalian target of rapamycin (mTOR) activation (20). Furthermore, autophagy, a process which is governs the degradation of misfolded proteins and damaged organelles, is important for normal placental developmental activities, such as invasion and vascular remodeling of extravillous trophoblasts (EVT). Studies on HTR8/SVneo cells showed that mTOR signaling also plays a role in regulating autophagy *via* the modulation of Beclin1 and synaptosome associated protein 29 (SNAP29) O-GlcNAcylation (21). Enhanced autophagy levels have been observed in human and mouse placentas exposed to HIP, as well as trophoblast cells in high-glucose environments (22–24). With regard to HIP, it is worth studying the exact function of the O-GlcNAcylation-associated regulation of autophagy in placental development.

Placental OGT and OGA expression levels both affect placental development; however, maternal stress seems to be the pivotal regulator of OGT and is more critical for fetal neurodevelopment, rather than hyperglycemia (13, 25–27). And, as an X-linked gene, placental OGT levels and its biochemical marker, O-GlcNAcylation are higher in females than in males. Male fetuses are associated with an increased risk of GDM in the mother (28, 29), and there might be an O-GlcNAcylation-related sexual dimorphism in the placental response to maternal hyperglycemia. In contrast, the regulation of OGA expression is more associated with glycemia. Dela Justina et al. (8) observed that increased O-GlcNAcylation accumulation in placental tissue exposed to severe hyperglycemia might contribute to an increased placental index and morphometric alterations, which could be associated with placental dysfunction. Although there were no changes in OGT expression in all groups, OGA expression was augmented in placentas from the mild hyperglycemic group and reduced in placentas from hyperglycemic rats. This might be a biological compensation phenomenon as a result of being confronted with a mounting supply of glucose through HBP flux. Yang et al. (30) proposed that OGA deletion suppresses hypoxia-inducible factor-1α (HIF-1α) stabilization and the transcription of its target genes, leading to impaired placental vasculogenesis and consequent disorders in fetal growth and development. The possible mechanism of O-GlcNAcylation and OGT in the translation and stabilization of HIF-1α has been studied in cancer cells. It was observed that an increased level of O-GlcNAcylation and the overexpression of OGT reduced α-ketoglutarate, which assists hydroxylation and the degradation of HIF-1α (31). Moreover, unbalanced O-GlcNAcylation levels favor endothelial dysfunction in uterine arteries, which is important for uteroplacental circulation and this is partly modulated by OGT (32). These results might partly explain the structural and functional immaturity of placentas exposed to hyperglycemia and its effect on maternal-fetal interactions (33).

In addition to its influence on placenta, O-GlcNAcylation can directly affect embryonic development, including the regulation of oocyte meiotic division, embryo implantation and the survival and differentiation process of embryonic pluripotent stem cells. Besides, O-GlcNAcylation-related excessive induction of reactive oxygen species (ROS) and subsequent oxidative stress leads to embryo DNA damage, mitochondrial instability, and cell apoptosis (34–36). A recent study also emphasized that the O-GlcNAc-dependent regulatory pathway is important for the DNA damage response required to maintain homeostasis in embryonic stem cells (37). Further, Muha et al. (38) proposed that the loss of OGA catalytic activity leads to widespread organ defects in mouse embryogenesis. Researchers have suggested that dysregulation of HBP and O-GlcNAcylation are major contributors toward the embryotoxic effects of hyperglycemia in early pregnancy (39). Another study also suggested that increased O-GlcNAcylation in metabolically compromised pregnancies, such as HIP, could be the underlying cause of defective neurodevelopmental outcomes (40).

## Nutrient Sensing

The placenta contains an array of nutrient-sensing signaling pathways. Of these nutrient sensors, mTOR and AMP-activated protein kinase (AMPK) play a key role (41). Their involvement in placental O-GlcNAcylation is responsible for placental development and glucose and amino acid transport (42) (**Figure 1**).

The atypical serine/threonine kinase mTOR is part of two complexes with distinct functions and structures: mTOR complex 1 (mTORC1) and mTORC2 (43). mTORC1 is highly expressed in trophoblast cells (44). mTOR plays an important role in controlling trophoblast cell growth, proliferation, syncytialization and macropinocytosis (45). mTOR activity is regulated by the concentration of glucose, amino acids, and insulin, and is mediated by the PI3K/AKT signaling pathway. It stimulates cell growth through the phosphorylation of tuberous sclerosis complex 2 (TSC2), a negative regulator of mTORC1, and activation of Ras homolog enriched in brain (46, 47). mTORC1 promotes protein synthesis by directly phosphorylating the eukaryotic translation initiation factor 4E (eIF4E) binding protein 1 (4E-BP1) and ribosomal protein S6 kinase (p70S6K) (48). The activity of GSK3β, an enzyme that regulates glycogen synthesis, is inhibited by the activation of insulin-AKT signaling pathway, which executes diverse biological functions (49). Besides, GSK3 phosphorylation of TSC2 inhibits the mTOR signaling pathway and the regulation requires AMPK activity (50). Dynamic changes in the AMP: ATP ratio regulate the activation of AMPK. In addition to participating in a variety of cellular activities such as lipid metabolism, AMPK targets the mTORC1 pathway, which plays a direct/indirect inhibitory role (51). Several participants of the PI3K/AKT/mTOR signaling pathway have been found to be modified by O-GlcNAcylation, such as IRS-1, PI3K, AKT, AMPK, p70S6K, 4E-BP1, and GSK3β (52–54).

Increased mTOR activity and decreased AMPK activity can be observed in placentas exposed to HIP (55–57). In a variety of tissues including the placenta, it has been proven that the expression, localization and activation of the key enzymes of O-GlcNAcylation are regulated by these nutrient-sensing signaling pathways. Studies on cardiomyocytes demonstrated that GFAT can be directly phosphorylated by AMPK, thereby reducing its activity and lowering O-GlcNAcylation levels (58). The localization, expression, and substrate specificity of OGT are regulated by AMPK, which is highly dependent on various factors such as the physiological/pathological status and cell types. In several pathologies, O-GlcNAcylation levels are reduced by AMPK activation to prevent adverse effects (59). Moreover, in Human HepG2 cells, it was observed that insulin stimulates the expression and activity of OGT and promotes its targeting to membranes which is dependent on activation of the PI3K pathway (60). Additionally, Kelly et al. (61) recently proved that the inhibition of mTOR signaling decreases the levels of OGT in the human placenta and affects development of the fetal brain. It was also proven in mouse brains that OGT is a substrate of GSK3β and that the phosphorylation of OGT by GSK3β increases OGT activity (62).

In contrast, OGT also acts as a nutrient sensor and regulates diverse cellular signaling pathways based on the metabolic status of cells by sensing glucose levels *via* UDP-GlcNAc concentrations and responding by dynamically O-GlcNAcylating proteins (63). Few studies have investigated the direct effects of O-GlcNAcylation on the PI3K/AKT/mTOR signaling pathway and its subsequent biological effects under physiological conditions. Under different disease states, O-GlcNAcylation has different activation/suppression effects on this signaling pathway (53, 54). Studies in the pancreas, liver, and skeletal muscle under diabetic conditions show that increased O-GlcNAcylation downregulates AKT and IRS-1 activity and inhibits the IRS-1/PI3K interaction. This leads to pancreatic β cell apoptosis, reduced glucose absorption through the downregulation of insulin-stimulated translocation of glucose transporter 4 (GLUT4) to the plasma membrane, and decreased gluconeogenesis through the regulation of GSK3β, which in turn contributes to blood glucose retention (64–69). The expression and activity of GLUTs in the placenta, which mediates maternal-fetal glucose transport, are also changed in HIP, but the influence of hyperglycemia has not been definitively concluded (70). Whereas GLUT1 was identified as the primary transporter in the placenta, James-Allan et al. demonstrated that (71) maternal insulin promotes GLUT4 trafficking to the fetal-facing basal plasma membrane of the STB. Moreover, during the entire process of gestation, the increase in the expression of GLUT4 meets the increased fetal nutrient demand and supports fetal growth. Further studies on skeletal muscle and adipose tissue proposed that GLUT4 could be directly O-GlcNAc modified, which might alter the translocation and transporter ability of GLUT4 (72). And Buller et al. found that basal glucose uptake and GLUT1 expression in rat LEF cell lines are inhibited by GSK3/TSC2/mTOR pathway (73). Whether O-GlcNAcylation can influence glucose uptake *via* direct modifications or the indirect regulation of GLUTs needs further investigation. The increase in protein O-GlcNAcylation in target tissues of diabetic patients might contribute to the maintenance of the pathological status of PI3K/AKT-mediated insulin resistance and could explain diabetic complications and adverse pregnancy outcomes (3, 74). O-GlcNAcylation of proteins could enhance the sensitivity of the PI3K/AKT/mTOR signaling pathway to nutrients. In addition, metformin, a potentially effective drug that might improve pregnancy outcomes for HIP, has been proposed to causes the upstream activation of AMPK, resulting in the inhibition of mTOR signaling in the placenta (75–79). It has been proven that metformin reduces the levels of OGT and O-GlcNAcylation and reverses the decreased phosphorylation level of AMPK cause by O-GlcNAc modification in cervical cancer cells. Therefore, further exploration of the possible O-GlcNAcylation-related mechanisms of metformin treatment in placentas exposed to hyperglycemia is required (80).

There seems to be a complex dynamic relationship between these three pathways, and their dynamic changes and interactions may explain the changes in placental nutrient transport in the presence of HIP.

## Inflammatory Reactions

Hyperglycemia leads to increased expression of pro-inflammatory cytokines, such as IL-6 and TNF-α, which impairs placental functions (81). The transcriptional activity of NF-κB, a nuclear factor inducing the expression of these proinflammatory cytokines, is regulated not only by phosphorylation and acetylation, but also by site-specific O-GlcNAcylation (82). Studies on the placenta of hyperglycemic rats show that non-classical activation of NF-κB is elicited by O-GlcNAcylation and that the p65 subunit is the main target for O-GlcNAcylation. After O-GlcNAcylation, NF-κB showed higher nuclear translocation and transcriptional activity, which may explain why NF-κB activity increases sustainably under hyperglycemic conditions (82, 83). In addition to the O-GlcNAcylation of NF-κB, Pathak et al. (84) determined that the activation of transforming growth factor (TGF)-β-activated kinase 1 (TAK1) needs the O-GlcNAcylation of TAK1-binding protein 1 (TAB1) to activate NF-κB and finally lead to the production of IL-6 and TNF-α in IL-1R HEK293 cells. However, O-GlcNAcylation can also be a negative regulator of NF-κB activity. According to a study in rat aortic smooth muscle cells, O-GlcNAc modification of NF-κB p65 inhibited TNF-α-induced inflammatory mediator expression (85).

Currently, studies associated with O-GlcNAcylation of transcription factors, especially those related to inflammation are limited. A study on cardiac fibrosis caused by diabetes mellitus (86) revealed that hyperglycemia enhanced O-GlcNAcylation of transcription factor Sp1. This modification increased its transcriptional activity, and promoted the expression of transforming growth factor β1 (TGF-β1) and fibrosis-related proteins such as collagen in cardiac fibroblasts. In the placenta, O-GlcNAcylation of Sp1 possibly interrupted the interaction of Sp1 with its cooperative factor to reduce its transcription (87).

Moreover, macrophages, called Hofbauer cells in the placenta, play key roles in chronic inflammatory processes, and long-term exposure to hyperglycemia causes macrophages to exhibit a pro-inflammatory phenotype (88). It has been recently shown in mouse bone marrow-derived macrophages (BMMs) that enhanced UDP-GlcNAc generation caused by increased HBP activity is a trait of M2 macrophages (89). However, there has been no specific study of O-GlcNAcylation in Hofbauer cells to date. Further, the few studies using different macrophage cell models that have evaluated the effect of O-GlcNAcylation on macrophage function report conflicting results. One study reported that O-GlcNAcylation promotes antiviral innate immunity and inflammatory responses in BMMs (90). Another study based on THP-1 cells and mouse peritoneal macrophages revealed that O-GlcNAcylation suppresses innate immune activation and necroptosis of macrophages (91). Additionally, O-GlcNAcylation was also proposed to attenuate inflammatory processes in macrophages induced by LPS which was observed in RAW264.7 cells, BMMs and peritoneal mouse macrophages, as well as human monocyte-derived macrophages (92). Yang et al. (93) indicated that overnutrition stimulates O-GlcNAc signaling in macrophages of a mouse model of diet-induced obesity. Further, the activation of O-GlcNAc signaling has a suppressive effect on macrophage proinflammatory activation by restraining mTORC1/S6K1 signaling, which contributes to whole-body metabolic homeostasis. These conflicting observations in macrophages might be related to tissue residency or M1/M2 polarization, and prompt further research on Hofbauer cells is required.

O-GlcNAcylation may be a two-sided modulator of inflammation (94–96). Transcription factors and functional proteins may be modified in different cell types, stimulation conditions, and nutritional states, which may affect their activities and initiate pro-inflammatory or anti-inflammatory functions. The specific role of O-GlcNAcylation in HIP requires further exploration.

## Conclusions

In summary, O-GlcNAcylation in the placental tissues of women with HIP plays an important role in placental development, nutrition sensing, and inflammatory response, and influences near- and long-term development of offspring. However, there are only a few relevant studies on the influence of O-GlcNAcylation on placental function. It is a process that has not been fully understood, particularly with regard to the regulation of transcription factors, intracellular signal transduction, and epigenetic modifications. As techniques to identify O-GlcNAcylation are increasingly being developed (97–101), further localization and quantitative analyses of O-GlcNAcylation in placental tissues exposed to hyperglycemia are required. This will facilitate the analysis of the effect of O-GlcNAcylation on the biological functions of placenta, as well as to understand the mechanistic details of the effect of maternal hyperglycemia on the development of offspring, particularly in relation to abnormalities in maternal-fetal nutrition transport and metabolism.

## Author Contributions

JN and HY wrote the manuscript. All authors contributed to the article and approved the submitted version.

## Funding

This work was supported by the National Natural Science Foundation of China (81830044).

## Conflict of Interest

The authors declare that the research was conducted in the absence of any commercial or financial relationships that could be construed as a potential conflict of interest.
